# Impact of Social Network Size and Contact Frequency on Resilience in Community-Dwelling Healthy Older Adults Living Alone in the Republic of Korea

**DOI:** 10.3390/ijerph18116061

**Published:** 2021-06-04

**Authors:** Sangmi Park, Tae-Hui Kim, Tae-Rim Eom

**Affiliations:** 1 Department of Occupational Therapy, Yonsei University, Wonju 26493, Gangwon-do, Korea; sangmipark@yonsei.ac.kr; 2 Department of Psychiatry, Yonsei University Wonju College of Medicine, Wonju 26426, Gangwon-do, Korea; 3 Health Insurance Research Institute, National Health Insurance Service, Wonju 26464, Gangwon-do, Korea; taerim0923@naver.com

**Keywords:** community health services, independent living, public health, preventive medicine, social participation

## Abstract

The aim of this study was to investigate the characteristics of social health and its association with resilience among older adults living alone excluded from the public care service due to their relatively good health. For this cross-sectional study, we surveyed older adults aged between 65 and 80 years using questionnaires to measure the social health status and levels of resilience of the participants. We conducted a hierarchical regression analysis to confirm the association between resilience and social network. Finally, data from 266 community-dwelling older adults were analyzed. We discovered that participants had social networks with a mean score on the Lubben Social Network Scale 18.13 ± 7.98, which means they were socially isolated. The network size (standardized β = −0.149, *p* < 0.05) and contact frequency (standardized β = 0.136, *p* < 0.05) correlated positively with higher levels of resilience. A hierarchical model accounted for 48.0% of the variance in resilience. The results suggested that interventions by the public health service to protect social health are needed for older adults living alone even when they are physically, emotionally, and cognitively healthy. In addition, smaller network size and higher frequency of contacts may be considered to strengthen resilience, which is a protective factor in social health.

## 1. Introduction

The burden of caring for elderly parents has been transferred from offspring to the public sector in the Republic of Korea [1,2,3]. This transition began with industrialization, urbanization, and nuclearization of family, and finally affected the traditional family values of supporting older parents. In addition, a rapid increase of aging population due to longer life span and continuous decline in birthrate triggered social issues, such as increasing socioeconomic care burdens caused by the increase in the older adults living alone or with dementia. Social isolation, characterized by living alone, is related to the risk of dementia, and the need to promote social networks is also suggested to prevent dementia [4]. Community care has been suggested for older adults living alone due to their higher rate of unsatisfied needs in social relationships or social participation as well as economic status and health conditions, compared with older adults living with family or housemates [3]. In 2007, the Ministry of Health and Welfare in South Korea established the Comprehensive Support Center for the Elderly Living Alone to administer community organizations that provide care and support services for older adults living alone [5,6]. Since then, various public care projects have been implemented by community organizations nationwide, such as the home helper dispatch service, the direct care worker dispatch service, and the emergency and safety care service [7,8].

As of 2019, there were 1.47 million senior citizens living alone in South Korea, which include 350,000 receiving the aforementioned public care services [9,10]. The services comprise mainly of safety support, daily life education, such as health education or nutrition education, and domestic help, focusing on physical assistance [7,8]. These services address the lower levels in the Maslow’s hierarchy of needs but do not include support for social participation or emotional support that correspond to an upper level of needs [11]. As the present public care service for senior citizens is biased towards physical health, the service partially addresses the concept of health, which encompasses physical, mental, and social aspects [12]. Therefore, with the present public care service, there could be unmet demands for mental health services [13]. The social health service also needs to be addressed as a public care service considering the fact that older adults perceive being connected with others as a component of health [14].

Resilience is also important for older adults living alone to protect their health status, as enhanced resilience helps older adults to overcome negative life events, which may cause health problems [15,16]. Resilience is defined as the personal ability to adjust to and overcome impacts of adverse events, which restores the stability of life [17,18]. Resilience in older adults is not likely to decline with ageing, but it is associated with various forms of social health, such as family network [15,19], social participation [20], or social support [21]. Given that older adults living alone are exposed to a higher risk of decrease in social participation, poor social support, or loneliness compared with older cohabiting individuals [22,23], studies investigating and protecting social health of those who live alone are needed. However, practical implications for protecting the social health of older adults living alone reported in previous studies are insufficient because the samples inadequately reflected the characteristics of older adults living alone. In addition, the result may be moderated by health issues, because the social network is connected to health issues, such as impaired cognitive function [24,25], physical disabilities [25], and depression [26,27].

In this study, we investigated the characteristics of social health in a healthy sample of older adults living alone to understand the status of social health. In addition, we analyzed the relationship between resilience and both network size and contact frequency to assess the importance of social network protection in healthy older adults living alone and to make suggestions for maintaining their social health.

## 2. Materials and Methods

### 2.1. Participants

We collaborated with the Comprehensive Support Center for the Elderly Living Alone to design a sampling method. We received a list of community-dwelling older adults who participated in the 2018 National survey of the elderly living alone and were excluded from the public care service because they were assessed as relatively wealthy or healthy people. The list included contact information of those who agreed to the use of their information for nonprofit services.

We conveniently selected two regions in the Gangwon province, one of the 16 administrative districts of South Korea, for our sample frame. Two regions, located close to the research team office, were selected to allow research team members to visit the participants easily if it was necessary. We selected a medium-sized city and a small town to include residents of both a city and a rural area. The sample frame consisted of 90.80% (*n* = 4756) of city-dwellers and 9.20% (*n* = 482) of town-dwellers. This rate reflected the rate of older adults living alone in cities and rural areas of South Korea. Considering the rate of refusal or drop-out, the contact information of 1013 older adults was extracted for this study by using the purposive sampling method.

We made calls to the people on the list and introduced the research. When the recipient was interested in participating in the study, after the phone call, we sent them a letter by post to explain the study. In a few days, we made a follow-up call to check if they had read the study explanation and to confirm their intent to participate in the study.

We recruited cognitively, physically, and emotionally healthy participants. The exclusion criteria were:

1. Aged older than 80
2. Scores less than −1.5 standard deviation in the Mini-Mental State Examination for Dementia Screening (MMSE-DS) [28]
3. Extremes in any dimension of mobility, self-care, usual activities, pain/discomfort, anxiety/depression in the Euro Quality of Life Questionnaire 5-Dimensional Classification, three-level version (EQ-5D-3L) [29]
4. Scores higher than 9 in the Korean version of Short form Geriatric Depression Scale (SGDS-K) [30]

### 2.2. Variables and Measurement

After confirming the participation intention over the phone, a trained investigator visited a participant for a screening test to confirm that the participant was without unhealthy symptoms in cognition, subjective health, or emotion. The screening tools for this study comprised the MMSE-DS [28], the EQ-5D-3L [29], and the SGDS-K [30]. This study was conducted in accordance with the Declaration of Helsinki and approved by the Institutional Review Board, and we received written consents from all participants before the survey. The same investigator who conducted the screening test collected the data through a one-on-one interview within a day. Visiting participants’ home was recommended to have an interview in a silent and settled environment. For a few participants who did not want to invite the investigator to their home, we prepared a meeting room located close to the research team office for interview.

#### 2.2.1. Independent Variables

##### Social Network

We used the Korean version of the Lubben Social Network Scale (LSNS) to assess participants’ social health status in this study [31]. Participants’ network size and contact frequency were measured with 6 of the 10 questions in the LSNS [31]. Social relationships are a part of social health. Measuring network size and contact frequency can be used to quantitatively evaluate the social health characteristics [32]. The number of close people or frequency of contacts have been commonly used to understand participants’ social network [24,33,34].

Generally, the result of the LSNS evaluates scores of 10 questions or subtotal scores according to social resources. The maximum score of the 10 questions is 50. A score higher than 31 indicates low risk of isolation, whereas scores between 26 and 30 suggest moderate risk for isolation, and a value between 21 and 25 indicates a high risk for isolation. Lower scores (<21) indicate isolation [35]. In this study, we used subtotal scores of network size and contact frequency in addition to a total score of the LSNS. Cronbach’s alpha for 10 questions in the present study was 0.724.

We measured the network size with four questions:

1. *“How many relatives do you see or hear from at least once a month?”*
2. *“How many relatives do you feel close to; i.e., how many of them do you feel at ease with when talking about private matters or calling for help?”*
3. *“Do you have any close friends; i.e., do you have any friends with whom you feel at ease or talk to about private matters or can call for help? If so, how many?”*
4. *“How many of these friends do you see or hear from at least once a month?”*

The response options included zero, one, two, three or four, five to eight, and nine or more. The maximum total score of the four questions was 20. Higher score indicated larger size of social network.

For contact frequency, we used two questions:

1. *“Tell me about the relative with whom you have the most contact. How often do you see or hear from that person?”*
2. *“Tell me about the friend with whom you have the most contact. How often do you see or hear from that person?”*

The answer options comprised less than monthly, monthly, a few times a month, weekly, a few times a week, daily or almost daily. The maximum score of the two questions was ten. A higher score suggested a higher frequency of social contact.

#### 2.2.2. Dependent Variable

##### Resilience

Multidimensional Individual and Interpersonal Resilience Measure (MIIRM) [36] was used to measure the resilience. The MIIRM was developed to measure older adults’ resilience and the tool contains 22 questions in 8 subcategories of self-efficacy, emotional regulation, emotional expression and communication, optimism, perceived economic and social resources, access to support network, relational accord, and spirituality and religiosity. The 22 questions consist of 9 questions with a 4-point scale, 11 questions with a 5-point scale, and 2 questions with a 10-point scale. The total score for the 22 questions ranges from 22 to 111. A higher score means a higher level of resilience. Concurrent validity of the MIIRM was confirmed with a correlation (r = 0.648, *p* < 0.001) between the MIIRM and the 10-item Connor–Davidson Resilience Scale (CD–RISC) [37]. In the development study, the internal consistency in the total score of MIIRM was represented by Cronbach’s α = 0.72 [36].

As we do not have a Korean version of MIIRM yet, all items in the MIIRM were translated into Korean by the research team to be applied in this study. We used 18 questions for analysis, excluding 4 questions related to access to support network to avoid improper correlation with independent variables. Cronbach’s alpha for the 18 questions was 0.781.

#### 2.2.3. Controlling Variables

##### Socio-Demographic Characteristics

We surveyed participant’s age, sex, years of living alone, years of education, existence of living children (0 = none, 1 = one or more than one), existence of living siblings (0 = none, 1 = one or more than one), having religion (e.g., Christianity, Catholicism, Buddhism, etc.) (0 = no, 1 = yes), and perceived socio-economic status (SES) using a questionnaire on a 5-point Likert scale (5 = very satisfied, 4 = satisfied, 3= moderate, 2 = unsatisfied, 1 = very unsatisfied), which we developed for this study.

##### Health-Related Variables

We investigated the perceived health issue in usual activities, the number of chronic diseases, cognitive function, and subjective health as health-related variables.

We used one question from the EQ-5D-3L [29] to assess the perceived health issue in usual activities. Because we used the EQ-5D-3L as a screening assessment and excluded those who answered “extreme problem” on any dimension, the participants’ answers were 1 (no problem) or 2 (some problems). Participants were asked how many chronic diseases they had from the list of stroke, heart disease, hypertension, diabetes, hyperlipidemia, pulmonary tuberculosis, and insomnia. Participants additionally described if they had other diseases, not in the list. The score of the MMSE-DS in the screening test was used to indicate cognitive health status: a higher level of cognitive function was associated with higher scores. Subjective health was measured using a 5-point Likert scale (5 = very healthy, 4 = healthy, 3 = moderate, 2 = unhealthy, and 1 = very unhealthy).

##### Emotion-Related Variables

Loneliness was measured using the Revised UCLA Loneliness Scale (RULS) [38]. Higher scores indicated higher levels of loneliness. Cronbach’s alpha for the 20 questions of the RULS in the present study was 0.873. The SGDS-K [30] score in the screening test was used. A higher score suggested higher level of depression.

### 2.3. Statistical Analysis

We used the SPSS Statistics software (SPSS Inc., Chicago, IL, USA), version 23, for all statistical analysis. We analyzed the data (Supplementary File S1) using a two-tailed test at a significance level of 0.05.

Descriptive analysis was used for general and social network characteristics of the sample. Before conducting the descriptive analysis to characterize the social network, we averaged the scores of network size (four questions) and contact frequency (two questions) separately to determine the number of people who the participants were in contact with and their contact frequency generally. Mean scores of network size and contact frequency were used as indices. We developed a conversion table to interpret indices based on the scales in the LSNS. Chi-square tests and independent t-tests were used to assess the difference in social network based on gender.

Analysis of variance was used to examine whether or not the level of resilience differed along with the difference in the network size or contact frequency. Scheffe’s post hoc test was used for comparison between groups.

We conducted hierarchical multiple regression to analyze the association between resilience and both network size and contact frequency. The variables validating their significant relationship with resilience were entered into a hierarchical regression model. We used Pearson correlation analysis for continuous variables and Spearman correlation analysis for ordinal variables. Independent t-tests were used to confirm the association between resilience and nominal variables. Socio-demographic variables were entered into model 1; health-related variables were entered into model 2; emotion-related variables were entered into model 3; and social network size and contact frequency were entered into model 4.

## 3. Results

In this study of 300 cases, 266 cases without any missing values were analyzed. The participants’ general characteristics and descriptive statistics of study variables are presented in Table 1. The participants’ average age was 71.85 years. The proportion of females was 59.0%.

Characteristics of older adults’ social network are presented in Table 2. Five male participants (1.9%) responded “none” to all questions about network size. Seventeen participants (female: 5, male: 12) (6.4%) answered “less than monthly” to all questions about contact frequency. There were four participants (1.5%) who answered “none” or “less than monthly” to all questions about network size and contact frequency. No statistical difference was found between male and females in chi-square tests (network size: χ^2^ = 4.84, df = 3, *p* = 0.184; contact frequency: χ^2^ = 6.85, df = 3, *p* = 0.077).

The average score of the LSNS (total score) was 18.13 (±7.98) and the score was lower than the cut-off score of 21, which suggests isolation [35]. The total score of females was significantly higher than that of males (t = 2.85, *p* = 0.005). While the score of network size was not significantly different between males and females (t = 0.36, *p* = 0.722), the score of contact frequency among females was significantly higher than that of males (t = 3.82, *p* = 0.001).

Mean scores of resilience by group according to network size and contact frequency are presented in Table 3. Participants with a larger network size tended to show higher resilience. Participants who reported their network size as three or more than three persons showed a significantly higher score of resilience than those who had less than one person (*p* = 0.021) and one to less than two persons (*p* = 0.037). Participants with more frequent contacts tended to exhibit higher scores of resilience. Participants who reported their contact frequency as weekly to less than a few times a week showed a significantly higher score of resilience than those who reported their contact frequency as less than a few times a month (*p* = 0.004). Participants who reported their contact frequency as a few times a week or more often showed a significantly higher score of resilience than those who reported contact frequency less than few times a month (*p* < 0.001) and a few times a month to less than weekly (*p* < 0.001).

The correlation coefficients of continuous and ordinal variables are presented in Table 4. Nominal variables, which were associated with resilience via t-tests, included existence of living children (t = −2.023, *p* = 0.044), religion (t = −4.502, *p* < 0.001), and perceived health problems in usual activities (t = 2.340, *p* = 0.020).

The results of hierarchical regression analysis are presented in Table 5. Religion, years of education, and subjective economic status were entered into model 1 including socio-demographical variables, excluding the variables of age (*p =* 0.344), gender (*p =* 0.326), years of living alone (*p =* 0.659), and existence of living siblings (*p* = 0.162), which were not significantly related to resilience. Subjective health and cognitive function were entered into model 2 among health-related variables, excluding the number of chronic diseases (*p =* 0.134). Loneliness and depression were entered into model 3 as emotion-related variables. Network size and contact frequency were entered into model 4.

The final model explained 48.0% of the variance in resilience (Table 5). The variance inflation factor (VIF) ranged from 1.088 to 1.794 in the final model and there was no multicollinearity.

Religiousness (β = 0.243, *p* < 0.01) and higher level of perceived SES (β = 185, *p* < 0.01) in model 1, higher level of subjective health (β = 0.173, *p* < 0.01) in model 2, and lower level of loneliness (β = −0.319, *p* < 0.01) and depression (β = −0.240, *p* < 0.01) in model 3 significantly correlated with higher levels of resilience. After controlling for socio-demographic characteristics, the health-related variables, and emotion-related variables, the network size (β = −0.149, *p* < 0.05) and contact frequency (β = 0.136, *p* < 0.05) showed statistically significant predictive impact on resilience in model 4. While network size was negatively correlated with resilience, contact frequency was positively correlated with resilience. The results showed that the participants with a smaller network size were likely to have higher levels of resilience, and the participants with more frequent social contacts were likely to have higher levels of resilience.

## 4. Discussion

In this study, we found that the LSNS score of our sample of community-dwelling older adults living alone excluded from the public care service due to their relatively good health conditions indicated that they were socially isolated. Their size of social network and frequency of social contacts were associated with resilience.

Given that the study sample comprised of community-dwelling older adults who were excluded from the public care service due to their relatively better health condition and socioeconomic status, their poor social health status was noteworthy. This result suggests the need for interventions to reduce social isolation, which is one of the serious health problems among older adults [39,40].

In this study, a positive association between resilience and contact frequency was confirmed by hierarchical regression analysis. Considering that resilience is linked to depression or mental health, it is likely to be in line with previous studies suggesting a positive aspect of the number of contacts. The positive effect of frequent contact with social network members for older adults may be supported by previous literature, such that frequent contacts with social network members had a positive effect on mental health and frequent contacts with friends or relatives indirectly ameliorated depressive symptoms [26,41].

However, the higher resilience associated with a larger network size based on a simple comparison of mean scores was not validated by the linear relationship. The association between smaller network size and higher levels of resilience in hierarchical regression modeling analysis could mean that community-dwelling older adults regarded emotional intensity of relationship as more important than larger size per se. Social support based on high-quality relationship is a preventative factor to overcome adversity [21]. Hence, resilience might be positively correlated with frequent contact with small networks of members who guaranteed higher quality in relationship. According to the socioemotional selectivity theory [42], as people age, older adults tend to be more motivated for participation which gives them higher emotional satisfaction. In the same vein, participants may prefer frequent contact with higher quality in a small group. These results may suggest that the two dimensions of network size and contact frequency should be considered together when we explore the characteristics of older adults’ social health.

As an alternative to helping older adults to reinitiate or maintain important social ties, joining a mutual support group could be feasible. Participating in a self-help group has a positive effect on psychological well-being by enhancing resilience through regular gathering and sharing of information or experience related to common interests or difficulties [43]. In this study, we found that the level of resilience was different depending on social network conditions. Considering the fact that improving resilience has a positive effect on wellbeing in later life [44,45], it is needed to protect older adults’ resilience via the public care service for their social health. To use or demonstrate the effect of self-help groups among community-dwelling older adults as a preventative approach, further studies are needed to determine the optimal size of group members or frequency of gathering and develop the specific framework such as online or in-person sessions.

Considerations for developing interventions for older adults to improve their resilience are not only the components of social network (i.e., size or contact frequency) but the purpose of the networking as well. Decreasing loneliness or depression, which showed relatively higher coefficients among the variables of the final model in our results, could be considered as the purpose of the self-help group. In addition, it is recommended to explore through further studies what type of network (e.g., conversation, task execution, or recreational activities) is suitable for older adults who need to develop new social ties. Also, we need to take into consideration the COVID-19 pandemic-accelerated exposure of older adults to the use of information and communication technology. Considering the spread of telehealth and the social atmosphere where people consider a none-contact service safer than an in-person service [46], it might be necessary to develop online interventions.

Our results have some limitations. First, the conversion table and average scores we used to determine the participants’ network size and contact frequency may be insufficient to identify the properties of the variables. We adopted this strategy to measure network size and contact frequency because of the difficulty in finding a validated tool focused on network size and contact frequency per se. Second, we could not control the unique characteristics of resilience in this cross-sectional study. The levels of individual resilience could be temporarily affected by individual adversities at the time of assessment. Although we recruited a homogeneous set of participants with respect to health condition and used validated tools to measure resilience, various level of individual resilience may limit the interpretation of the study results. Third, the convenience sampling in this study limits us to generalize the result for a population of older adults living alone in relatively good health conditions.

## 5. Conclusions

Healthy community-dwelling older adults living alone experienced social isolation, and their network size and contact frequency were related to their resilience. This study suggests that interventions addressing social health by the public health service are needed for older adults living alone even when they are physically and mentally healthy. In addition, protecting the present important social ties and enriching the network through frequent contact may be considered to strengthen resilience, which is a protective factor in social health.

## Figures and Tables

**Table 1:** General characteristics of participants and descriptive statistics (*n* = 266).

General Characteristics	*n* (%)	Mean (SD)
Age (year)		71.85 (4.02)
Years of living alone		17.49 (11.68)
Years of education		6.85 (4.30)
Number of chronic diseases		1.82 (1.30)
Gender (Female)	157 (59.0)	
Living children		
None	12 (4.5)	
More than one	254 (95.5)	
Living siblings		
None	40 (14.1)	
More than one	226 (85.9)	
Religion		
Not Religious	96 (36.1)	
Religious	170 (63.9)	
Perceived SES		
Very unsatisfied	66 (24.8)	
Unsatisfied	103 (38.7)	
Moderate	81 (30.5)	
Satisfied	12 (4.5)	
Very satisfied	4 (1.5)	
**Perceived health problems in usual activities**		
None	242 (91.0)	
Some	24 (9.0)	
Subjective health		
Very unhealthy	10 (3.8)	
Unhealthy	69 (25.9)	
Moderate	89 (33.5)	
Healthy	80 (30.1)	
Very healthy	18 (6.7)	
MMSE-DS		27.21 (2.36)
RULS		38.83 (10.74)
SGDS-K		3.43 (2.69)
Social Network		
Size		7.84 (3.84)
Frequency		5.48 (2.69)
MIIRM ^†^		63.63 (10.07)

SD = standard deviation, SES = socioeconomic status, MMSE-DS = Mini-Mental State Examination for Dementia Screening, RULS = Revised UCLA Loneliness Scale, SGDS-K = Korean version of Short-form Geriatric Depression Scale, MIIRM = Multidimensional Individual and Interpersonal Resilience Measure. †: Subtotal score of 18 items in MIIRM was used for analysis in this study. Mean and SD for the total score of the 22 items was 71.26 (11.26).

**Table 2:** Characteristics of participants’ social network (*n* = 266).

Characteristics	Total *n* (%)	Female *n* (%)	Male *n* (%)	χ^2^/t
Network size				
Less than 1 person	34 (12.8)	15 (9.6)	19 (17.4)	
1—Less than 2 persons	93 (35.0)	61 (38.9)	32 (29.4)	X^2^ = 4.84 df = 3 *p* = 0.184
2—Less than 3 persons	90 (33.8)	52 (33.1)	38 (34.9)	
3—More than 3 persons	49 (18.4)	29 (18.5)	20 (18.3)	
Contact frequency				
Less than a few times a month	56 (21.1)	26 (33.1)	30 (22.9)	X^2^ = 6.85 df = 3 *p* = 0.077
A few times a month–Less than weekly	67 (25.2)	38 (24.2)	29 (26.6)	
Weekly–Less than a few times a week	75 (28.2)	46 (29.3)	29 (26.6)	
A few times a week or more often	68 (25.6)	47 (29.9)	21 (19.3)	
LSNS scores (Mean ± SD)				
Total score (10 questions)	18.13 ± 7.98	19.27 ± 7.60	16.48 ± 8.26	t = 2.85 **
Network size (4 questions)	7.84 ± 3.84	7.91 ± 3.50	7.73 ± 4.29	t = 0.36 *p* = 0.72
Contact frequency (2 questions)	5.48 ± 2.69	5.92 ± 2.51	4.84 ± 2.82	t = 3.82 **

*n* = 266 (Female: 157, Male: 109), LSNS = Lubben Social Network Scale; Percentage (%) indicates the proportion in the column. ** *p* < 0.01.

**Table 3:** Difference in mean scores of resilience according to social network (*n* = 266).

Groups		*n*	Resilience		F	*p*	Post-Hoc
			Mean	SD			
Network size	Less than 1 person ^a^	34	60.22	10.02	4.56	0.004	a < d *, b < d *
	1—Less than 2 persons ^b^	93	62.04	8.65			
	2—Less than 3 persons ^c^	90	64.64	10.27			
	3—More than 3 persons ^d^	49	67.16	11.12			
Contact Frequency	Less than a few times a month ^e^	56	58.46	8.75	11.56	0.000	e < g **, e < h **, f < h **
	A few times a month–Less than weekly ^f^	67	62.17	9.37			
	Weekly–Less than a few times a week ^g^	75	64.65	9.03			
	A few times a week or more often ^h^	68	68.21	10.70			

* *p* < 0.05, ** *p* < 0.01. ^a^—Less than 1 person; ^b^—Less than 2 persons; ^c^—Less than 3 persons; ^d^—More than 3 persons; ^e^—Less than a few times a month; ^f^—A few times a month–Less than weekly; ^g^—Weekly–Less than a few times a week; ^h^—A few times a week or more often.

**Table 4:** Correlation analysis.

	Edu	SES	Cog	SH	Lon	Dep	Size	Freq	Res
Edu	1	0.164 **^,‡^	0.531 **^,†^	0.200 **^,‡^	−0.146 *^,†^	−0.049 ^†^	0.240 **^,†^	0.124 *^,†^	0.154 *^,†^
SES		1	0.070 ^‡^	0.313 **^,‡^	−0.222 **^,‡^	−0.265 **^,‡^	0.187 **^,‡^	0.135 *^,‡^	0.370 **^,‡^
Cog			1	0.188 **^,‡^	−0.165 **^,†^	−0.138 **^,†^	0.202 **^,†^	0.020 ^†^	0.164 **^,†^
SH				1	−0.224 **^,‡^	−0.376 **^,‡^	0.200 **^,‡^	0.085 ^‡^	0.402 **^,‡^
Lon					1	0.417 **^,†^	−0.515 **^,†^	−0.464 **^,†^	−0.483 **^,†^
Dep						1	−0.295 **^,†^	−0.202 **^,†^	−0.499 **^,†^
Size							1	0.493 **^,†^	0.241 **^,†^
Freq								1	0.332 **^,†^
Res									1

Edu = years of education, SES = perceived socioeconomic status, SH = subjective health, Cog = cognitive function, Lon = loneliness, Dep = depression, Size = size of social network, Freq = frequency of social contact, Res = resilience. * *p* < 0.05; ** *p* < 0.01; ^†^—*r* with Pearson correlation test; ^‡^—*r* with Spearman correlation test.

**Table 5:** Hierarchical regression analysis (Standardized beta coefficient).

Variables	Model 1	Model 2	Model 3	Model 4
Years of education	0.117 *	0.033	0.048	0.052
Existence of living children	0.077	0.063	−0.016	−0.025
Religion	0.245 **	0.251 **	0.254 **	0.243 **
Perceived SES	0.338 **	0.259 **	0.185 **	0.185 **
Perceived health problems in usual activities		−0.061	−0.082	−0.060
Cognitive function		0.081	0.009	0.029
Subjective health		0.271 **	0.156 **	0.173 **
Loneliness			−0.312 **	−0.319 **
Depression			−0.233 **	−0.240 **
Size of social network				−0.149 *
Frequency of social contact				0.136 *
Adjusted R^2^	0.212	0.290	0.465	0.480
R^2^ change	0.212	0.078	0.175	0.015
*F*	18.827 **	10.592 **	43.035 **	4.670 *

Dependent variable: resilience, SES = socioeconomic status, * *p* < 0.05, ** *p* < 0.01.

## Data Availability

The data presented in this study are available as supplementary material.

## Supplementary Materials

The following are available online at http://doi.org/10.5281/zenodo.4723552, File S1: The minimal dataset for analysis.
